# The proteome of neurofilament-containing protein aggregates in blood

**DOI:** 10.1016/j.bbrep.2018.04.010

**Published:** 2018-05-25

**Authors:** Rocco Adiutori, Johan Aarum, Irene Zubiri, Michael Bremang, Stephan Jung, Denise Sheer, Ian Pike, Andrea Malaspina

**Affiliations:** a Centre for Neuroscience and Trauma, Queen Mary University of London, Blizard Institute, Barts and The School of Medicine and Dentistry, London, United Kingdom; b Centre for Genomics and Child Health, Queen Mary University of London, Blizard Institute, Barts and The London School of Medicine and Dentistry, London, United Kingdom; c Proteome Sciences Plc, Hamilton House, Mabledon Place, London, United Kingdom; d ProteomeSciencesR&DGmbH&Co.KG, Frankfurt, Germany

**Keywords:** NCH, neurofilament-containing hetero-aggregates, Nf, neurofilaments, NfH, neurofilament heavy chain, NfL, neurofilament light chain, NfM, neurofilament medium chain, PPS, pooled plasma sample, SEP, Seprion PAD-beads, UC, ultracentrifugation, Neurofilaments, Protein aggregates, MS-based proteomics, Blood biomarkers, Ultracentrifugation, Seprion PAD-Beads

## Abstract

Protein aggregation in biofluids is a poorly understood phenomenon. Under normal physiological conditions, fluid-borne aggregates may contain plasma or cell proteins prone to aggregation. Recent observations suggest that neurofilaments (Nf), the building blocks of neurons and a biomarker of neurodegeneration, are included in high molecular weight complexes in circulation. The composition of these Nf-containing hetero-aggregates (NCH) may change in systemic or organ-specific pathologies, providing the basis to develop novel disease biomarkers. We have tested ultracentrifugation (UC) and a commercially available protein aggregate binder, Seprion PAD-Beads (SEP), for the enrichment of NCH from plasma of healthy individuals, and then characterised the Nf content of the aggregate fractions using gel electrophoresis and their proteome by mass spectrometry (MS). Western blot analysis of fractions obtained by UC showed that among Nf isoforms, neurofilament heavy chain (NfH) was found within SDS-stable high molecular weight aggregates. Shotgun proteomics of aggregates obtained with both extraction techniques identified mostly cell structural and to a lesser extent extra-cellular matrix proteins, while functional analysis revealed pathways involved in inflammatory response, phagosome and prion-like protein behaviour. UC aggregates were specifically enriched with proteins involved in endocrine, metabolic and cell-signalling regulation. We describe the proteome of neurofilament-containing aggregates isolated from healthy individuals biofluids using different extraction methods.

## Highlights

• NfH is part of high molecular weight protein complexes in blood from healthy controls.
• Proteome comparison of plasma aggregates enriched with different methodologies.
• Aggregates containing NfH may be potential blood biomarkersof neurodegeneration.

## 1 Introduction

The formation of assemblies of proteins that have lost their soluble state is a pathological hallmark of several neurodegenerative diseases [1], [2]. The confluence of proteins into aggregates may also occur physiologically as shown for the recruitment of RNA-binding proteins into stress granules [3], [4], [5], [6], [7]. In cells, the aggregation of misfolded proteins is usually kept in check by a quality control system, which operates through protein re-folding, autophagy and clearance by the proteasome [8], [9]. Extracellularly, a range of immune mediators may contribute to the clearance of misfolded proteins and of their aggregated forms [10]. It is proposed that in biological fluids, aggregate formation reflects the propensity of proteins to assemble naturally or can be experimentally induced under conditions of stress [11], [12], [13]. Depletion of albumin from human plasma, for example, leads to a significant increase in protein aggregation, particularly when heat and shear stress are applied [14]. Recently the presence of soluble sodium dodecyl sulphate (SDS) resistant protein aggregates has been reported in plasma from older adults and in significantly lower levels in plasma from younger individuals [15]. Loss of protein homeostasis and the increased rate of intra-cellular protein aggregation seem to be important hallmarks of aging [15]. Therefore, aggregates found in circulation may originate from senescent cell that have lost their functional integrity. Equally, an age-related failure of the control of protein homeostasis (i.e., proteostasis) may condition an increase of aggregation-prone proteins in fluid state and the formation of aggregates [15].

Separation of protein aggregates from fluids for quality assessment of biopharmaceutical formulations or for diagnostic purposes can be obtained by sedimentation-density analysis using ultracentrifugation (UC) or by extraction technologies that utilize solid-state binders. The Seprion PAD-beads (SEP) technology, for example, has been applied to the capture of proteins in different states of aggregation [16]. How different isolation methods compare with regards to the pool of specific aggregates found in the extraction products and their protein composition, particularly when biofluids are the source of the target particles, is not known.

Aggregates, inclusion bodies or aggregosomes described in neurodegenerative diseases contain neurofilaments (Nf), the building blocks of neurons [17], [18]. Nf are type IV intermediate filaments and one of the most abundant cytoskeletal component known to stabilize axons and maintain their size and functionality. Nf isoforms include neurofilament light (NfL, 61.5 kDa), medium (NfM, 102.5 kDa) and heavy (NfH, 112.5 kDa) chains. Post-translational modifications (PTMs), including phosphorylation and glycosylation at amino acids Serine and Threonine, affect Nf properties and size which increases to 70, 170 and 200 kDa for NfL, NfM and NfH, respectively [19], [20]. Nf isoforms self-assembly and interaction with other molecules depend on conformational changes and PTMs, which also condition their immunogenicity [21], [22], [23]. Nf are released in cerebrospinal fluid (CSF) and blood when neurons and axons degenerate, and changes of Nf levels are associated with the progression of several neurodegenerative disorders [22], [24], [25]. We have recently suggested that circulating Nf are also present in high molecular weight molecular complexes. Immunodetection of heavy chain Nf (NfH), for example, when tested in serial sample dilutions, lacks the linearity of the calibration curves seen with Nf recombinant proteins or light chain Nf (NfL), a property explained by the so-called *hook effect* [21]. This phenomenon may relate to NfH epitopes being masked due to sequestration into immunocomplexes or other molecular assemblies, which is disrupted by the dilution process [21]. These data suggest that the formation of Nf-containing hetero-aggregates (NCH) is possible in both tissues and fluids [22], [24]. Understanding Nf distribution between low order oligomers and higher order hetero-aggregates in biofluids has significant implications on their utility as biomarkers. In amyotrophic lateral sclerosis (ALS), an invariably fatal neurodegenerative disorder, Nf form heterogenous protein aggregates [26] and it is assumed that these are released, essentially intact, into the blood stream following cell death. The de-novo formation of circulating heteroaggregates due to a seeding effect of proteins like Nf in the fluid phase cannot be excluded. We hypothesise that neurofilaments may be released under both normal and pathological conditions as hetero-aggregates and that the content of these formations may differ between neurologically normal and diseased individuals. Hence defining the presence and content of NCH in normal individuals is a necessary step towards developing their utility as a new source of ALS biomarkers. Establishing protocols for NCH isolation and molecular characterization is therefore mandatory for any future use of NCH as disease biomarkers.

Here we studied circulating highly stable Nf-containing aggregates in plasma of neurologically healthy individuals using gel-based separation and described their protein composition. For the isolation of these aggregates from biological fluids, we have tested different conditions of ultracentrifugation including detergents and high salt concentrations and compared this approach to extraction obtained using aggregate capture binders (Seprion Ligand, SEP [27]). Liquid chromatography tandem mass spectrometry (LC-MS/MS) was then used to characterize the protein content of the complexes isolated by UC and SEP.

## 2 Methods

### 2.1 Plasma samples

Plasma samples from 6 healthy individuals with no known neurological disorders were pooled to be used in the enrichment methods. The selected individuals were aged between 51.2 and 62.9 years at the time of blood sampling. Neurofilament heavy chain (NfH) concentration ranged from 7.0 and 42.9 ng/ml (NfH levels were determined using immunodetection by sandwich ELISA as previously described by Lu et al. [22]). The pooled plasma sample (PPS) was divided in 1.1 ml aliquots and stored at − 80 °C. Ethical approval for recruitment, sampling and for the experimental procedures was obtained by the East London and The City Research Committee (09/H0703/27).

### 2.2 Total protein quantification

Total protein quantification was carried out using Pierce BCA Protein Assay Kit (Thermo Fisher Scientific) or Bio-Rad Protein Assay Kit (Bio-Rad) according to compatibility with reagents used in the protocol.

### 2.3 Western blotting

HiMark™ pre-stained Protein Marker (Fisher Scientific UK Ltd) and samples were loaded into 3–8% Tris-Acetate gels (Fisher Scientific UK Ltd) and, after electrophoresis, proteins and marker were transferred onto a nitrocellulose or polyvinylidene fluoride (PVDF) membrane (Fisher Scientific UK Ltd). The membrane was blocked with 5% skimmed milk in TBS 0.1% Tween-20 buffer at room temperature for 1 h. Overnight incubation was performed with primary antibody at 4 °C followed by incubation with secondary antibody for 1 h at room temperature, with membrane washes between steps using TBS 0.1% Tween-20. The membrane was then incubated with enhanced chemiluminescence substrate (ECL) and visualised using a BioRad Chemi-Doc system. For serial probing of the same membrane with different antibodies, stripping with ReBlot Plus Mild Antibody Stripping Solution (Millipore) for 15 min was performed.

### 2.4 Nf expression in pooled plasma samples

To first evaluate the presence of neurofilaments (Nf)-containing high molecular weight protein aggregates in pooled plasma samples (PPS) by western blot (WB), aliquots were first filtered twice with Amicon filters 100 K (Millipore). In this step, NfL isoform (~70 kDa) was likely to be retained by the filter. Different conditions known to solubilise plasma aggregates were also employed as previously reported and detailed hereafter [21]. Pooled plasma samples aliquots prepared for NfH, NfM and NfL analysis were divided into three fractions and processed as follows: 1) pre-treatment with 0.5 M urea and Barb_2_EDTA buffer for 1 h at RT, 2) dilution 1:1 with Barb_2_EDTA Buffer for 1 h at RT and 3) left untreated at + 4 °C.

### 2.5 Antibodies

The following antibodies and relative dilutions (in brackets) were used in this study: anti-Neurofilaments light (NfL; 1:1000) (clone EP675Y, Rabbit, Millipore), anti-Neurofilament Medium (NfM; 1:1000) (AB1987, Rabbit, Millipore), anti-Neurofilament heavy (NfH; 1:1000) (N4142, Rabbit, Sigma-Aldrich), Swine Anti-Rabbit Immunoglobulins (1:50,000) (P021702-2, DAKO).

### 2.6 Protein aggregate enrichment methods

#### 2.6.1 Ultracentrifugation

To identify the best conditions for enrichment of NCH using ultracentrifugation (UC), we tested different detergents (SDS, Triton X-100, Sarkosyl) at either 0.5% or 2% and different NaCl concentrations (0.5 M, 1 M, 1.5 M) for salting in (Supplementary Table 1). The best conditions were selected based on Nf detection, both in native and within high MW forms. Total protein concentration was measured to evaluate sample protein enrichment (Supplementary Fig. 1). 111 µl of 20% Triton X-100 was added to 1 ml of PPS (final concentration 2% Triton X-100) and incubated in agitation for 10 min at RT. Centrifugation at 21,000*g* for 15 min was performed at RT and supernatant was collected for ultracentrifugation (UC). At this stage, 800 µl Sucrose Cushion (1 M sucrose, 50 mM Tris-HCl, 1 mM EDTA and 2% Triton X-100) was added into each UC tube (Open-Top/Self-Seal, PA, 8 × 51 mm, Science Services GmbH) together with 500 µl of pre-cleared plasma onto the cushion. After balancing the tubes, UC was undertaken using a Sorvall Discovery 100SE, equipped with a TFT 80.2 rotor for 2 h at 50,000 rpm, 4 °C. The supernatant was discarded and pellet re-suspended with PBS, 1.5 M NaCl and vortexed for 30 s for washing. After an additional UC step of 40 min at 50,000 rpm (4 °C), the final pellet was re-suspended in 100 µl PBS for western blotting or 100 µl 8 M urea for MS analysis and stored at − 80 °C.

#### 2.6.2 Seprion PAD-beads (Microsens Biotechnologies, UK)

The Seprion PAD-beads (SEP) isolation method is based on the proprietary ligand effect in retaining protein aggregates [27]. To evaluate optimal binding conditions, we tested different volumes of samples and reagents in the final mix (Table 1) and used WB for Nf detection. Different loading volumes of PPS were mixed with 200 µl capture buffer and 100 µl of Seprion reagent. Seprion magnetic PAD-beads were re-suspended and 100 µl transferred into the reaction mixture, followed by 30-min incubation in agitation at RT. Beads were washed at 95 °C for 10 min and sample eluted with 4 × loading buffer (Fisher Scientific UK Ltd) and 50 mM DTT. The final SEP aggregate-containing fractions were not quantified for total protein concentration as the loading buffer was not compatible with the protein quantification methods reported above.

**Table 1:** Conditions for aggregate enrichment by Seprion Magnetic PAD-Beads: volumes of samples and reagents; CB: Capture Buffer; SR: Seprion-PAD Reagent.

	**Seprion Enrichment Condition**						
**Reagent**	**1**	**2**	**3**	**4**	**5**	**6**	**7**
**Sample [µl]**	800	400	400	400	400	400	200
**CB [µl]**	200	400	200	200	400	400	200
**Water [µl]**	0	0	200	200	0	0	400
**SR [µl]**	100	200	100	100	100	100	100
**Beads [µl]**	100	200	200	100	200	100	100

### 2.7 Proteomics analysis by LC-MS/MS

SDS-PAGE was performed with aggregate-containing samples obtained using UC and SEP as described above. The gel was stained with Imperial Protein Stain for 1 h at RT, washed overnight with ddH_2_O and then cut into 15 different fractions according to the band profile. Each gel fraction was reduced with DTT and alkylated with iodoacetamide, then de-stained and subjected to in-gel trypsin digestion. Peptides were extracted and freeze-dried for subsequent LC-MS/MS. Products of Ultracentrifugation and Seprion-based extraction were re-suspended, cleaned via zip-tip before injection. Qualitative analysis was performed using Thermo Scientific™ OrbitrapVelos Pro mass spectrometer coupled to an EASY-nLC 1000 (Proxeon) system. Samples were resuspended in 10 µl of 2% ACN/0.1% formic acid (FA), and then 5 µl was injected onto a 75 µm × 2 cm nanoViper C18 Acclaim PepMap100 precolumn (3 μmparticle size, 100 Å pore size; P/N 164705; Thermo Scientific) with an additional sample loading volume of 12 µl of 0.1% FA in H_2_O using the Thermo Scientific EASY-nLC 1000 system. Peptides were separated at a flow rate of 250 nl/min and eluted from the column over a 60-min gradient starting with 0.1% FA in ACN (5–30% over 50 min, then 30–80% between 50 and 54 min, continuing at 80% up to 58 min) through a 75 µm × 50 cm PepMap RSLC analytical column at 40 °C (2 µm particle size, 100 Å pore size; P/N ES803; Thermo). After Electrospray Ionisation, MS spectra ranging from 350 to 1800 *m/z* values were acquired in the Orbitrap at 30k resolution and the 20 most intense ions with a minimal required signal of 5000 were subjected to MS/MS by rapid CID fragmentation in the ion trap. Protein identification was carried out with a Thermo Scientific Proteome Discoverer 1.4.

The number of spectra acquired from the instrument and ID rate (Peptide Spectrum Matches (PSMs)/total spectra) were used to verify the even loading of test-samples and PSMs performances to test overall coverage within the sub-groups of proteins identified.

### 2.8 Bioinformatic analysis

The aggregates protein profile was analysed using Protein ANalysisTHrough Evolutionary Relationships (PANTHER, version 12.0 http://pantherdb.org/), a classification system in GO terms for large-scale data mining [28], and Webgestalt (WEB-based Gene SeTAnaLysis Toolkit, 2017 http://webgestalt.org/option.php), a functional enrichment analysis tool [29]. Bioinformatics analysis was performed to study the UC and SEP fractions and compare the range of aggregate-containing proteins the two methods had captured. With a focus on the origin of the protein pools, analysis was initially performed to dissect features related to Cellular Component (CC). KEGG pathway enrichment was also undertaken to identify pathways and biological features in the protein mix within the fractions isolated by UC and SEP. False Discovery Rate (FDR) was chosen as the statistical parameter to select the most relevant KEGG pathways [30].

## 3 Results

### 3.1 Neurofilaments are present in plasma

**Fig. 1:**
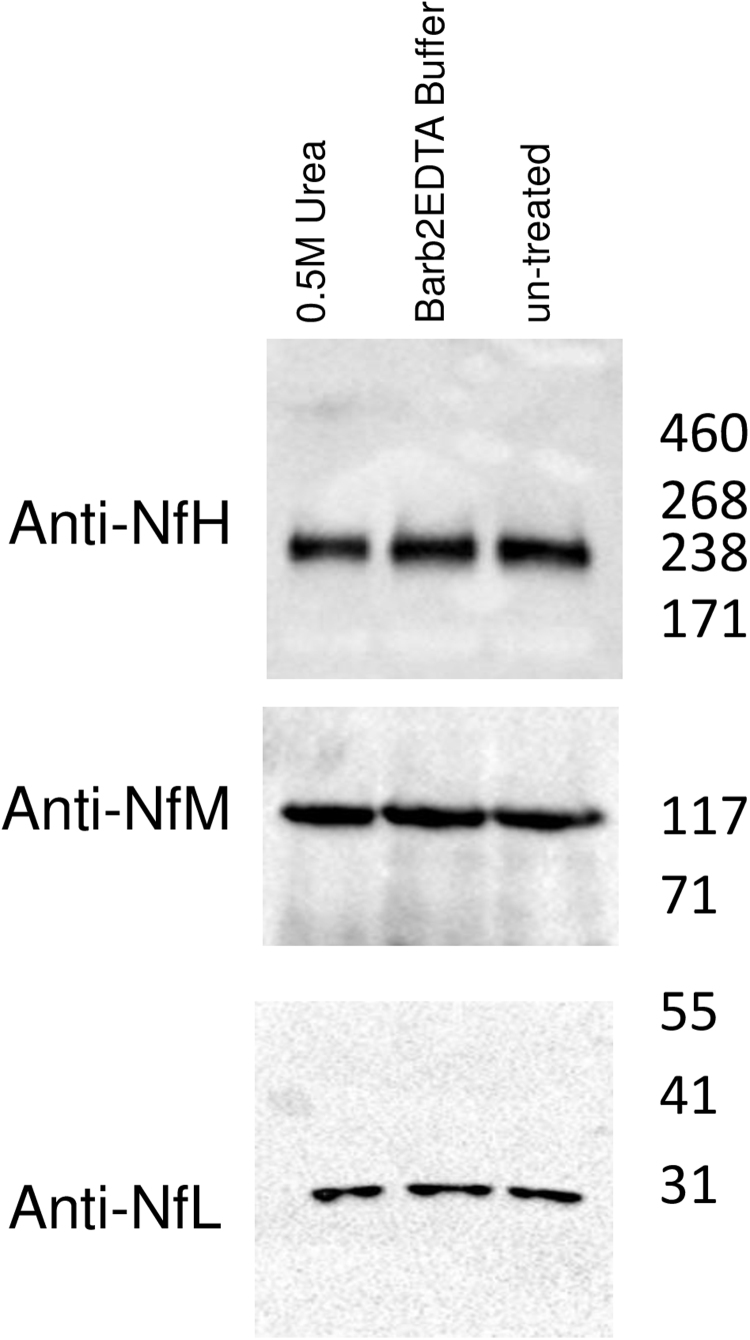
Western blot analysis of pooled plasma sample (PPS) using anti-neurofilament-High (NfH), anti-Medium (NfM) and anti-Low (NfL) antibodies after filtration with Amicon 100 filters (100 kDa molecular cut-off). Lanes contain in order from left to right: PPS subjected to pre-treatment with 0.5 M urea and Barb2EDTA buffer for 1 h at RT, PPS diluted 1:1 with Barb2EDTA Buffer for 1 h at RT and untreated PPS kept at + 4 °C. The blots show the three neurofilament proteins, with NfL appearing at approx. 30 kDa, NfM at 117 kDa and NfH at 238 kDa. No bands with molecular weight higher than the expected sizes, indicative of stable Nf-containing aggregates, were detected.

To minimize sample variability, we pooled plasma samples from six healthy donors. The presence of Nf, both free and within aggregates, was investigated in this pooled plasma sample (PPS) following ultrafiltration (repeated twice) with a 100 kDa cut-off filter, to concentrate Nf species in plasma and increase their detection by western blot (WB). The retained fraction was subjected to western blot (WB) as previously described [21]. One of the samples was treated with 0.5 M urea to dissolve potential aggregates. Fig. 1 shows the presence of NfH and NfM in the pooled plasma samples (PPS) at the expected molecular weights (MW). A lower than expected NfL-reactive band was also detected at approximately 30 kDa (expected MW~70 kDa), possibly related to the release of a NfL fragment from protein complexes retained by the filter, by the SDS detergent used in the WB procedure. No SDS-stable immunoreactive bands above the expected Nf MW's were identified, irrespective of the use of urea, and Nf bands intensity and MW appeared comparable across conditions. We show that all three Nf isoforms are present in plasma, and possibly, at least for NfL, as part of high-molecular weight assemblies (> 100 kDa, based on ultrafiltration). To investigate this, we proceeded to enrichment of high molecular weight assemblies by sedimentation analysis using ultracentrifugation.

### 3.2 Conditions of enrichment by ultracentrifugation

We initially compared various conditions for ultracentrifugation by altering the amount of salt (NaCl) and detergents, e.g. SDS and Triton X-100 (Supplementary Material Table 1). Pre-treatment of samples with a final concentration of 2% Triton X-100, centrifugation with a 1 M sucrose cushion and pellet washing using 1.5 M NaCl was associated with a clear detection of NfL, NfM and NfH by WB (Supplementary Material, Fig. 1). Under these conditions, NfH was consistently present at high MW (more than 200 kDa), while NfM and NfL both migrated at the same MW (117 kDa and 30 kDa respectively) reported for Nf detection in un-processed PPS (Fig. 1; Supplementary Material, Fig. 1). The detection of high MW, SDS-stable, NfH-containing bands in the extraction products is in keeping with the presence of circulating, NfH-containing protein aggregates.

### 3.3 Enrichment of neurofilament-containing aggregates by Seprion PAD-beads (SEP)

**Fig. 2:**
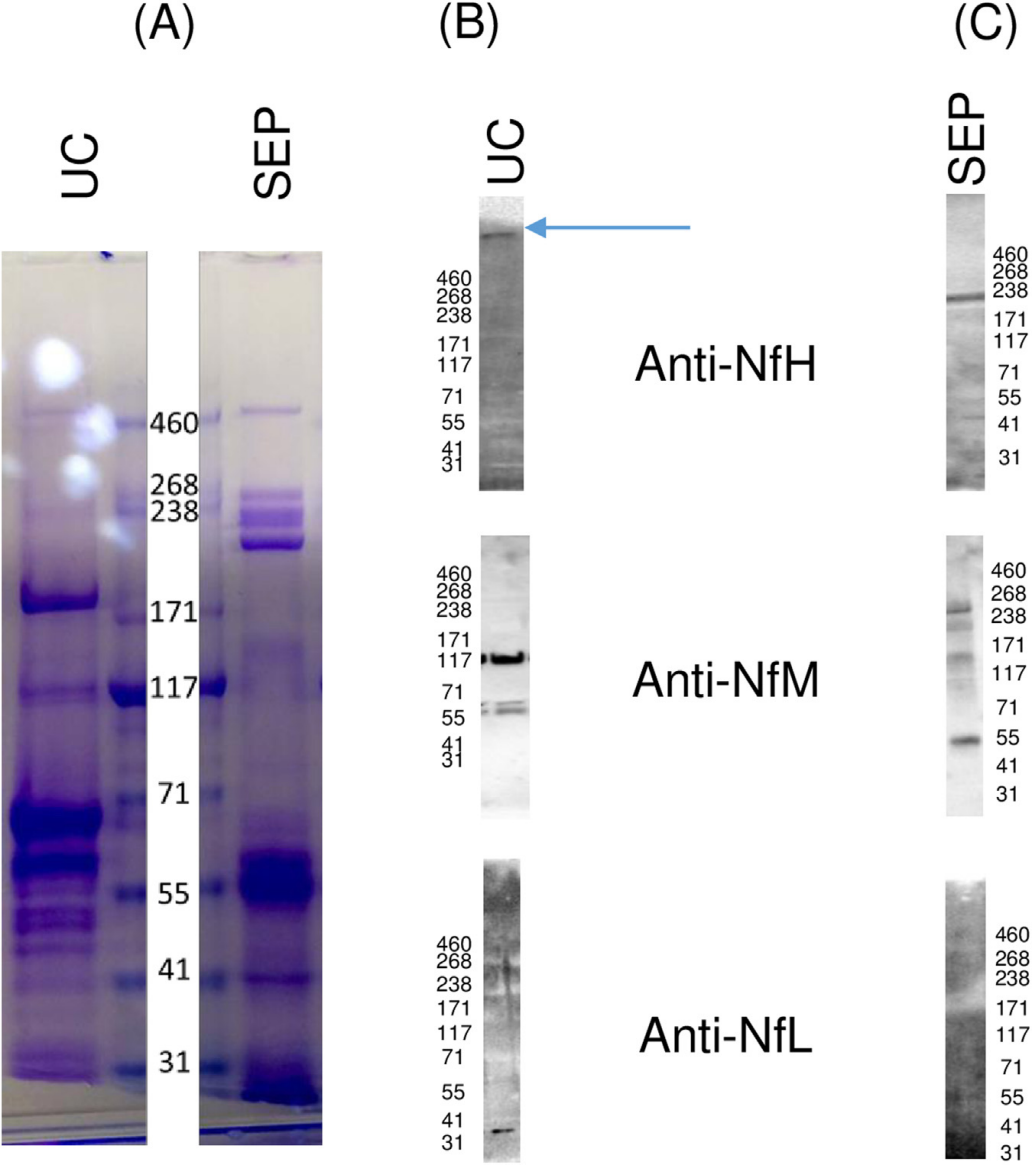
Comparison of aggregate fractions extracted from pooled plasma samples (PPS) using ultracentrifugation (UC) and Seprion extraction Pads (SEP). (A): Comassie-stained SDS-PAGE gel showing the different protein profiles of the enriched fractions extracted using the two methodologies. (B-C): western Blots analysis showing presence of Nf within the aggregates fractions obtained through UC (B) and SEP (C). Arrow highlights the presence of anti-NfH-containing high MW band.

Having established a method to isolate Nf-containing protein aggregates from plasma by UC, we next compared it to aggregate-isolation with SEP [31], using the same pooled plasma samples (PPS) as test-material under condition 1, starting with 800 µl of PPS (Table 1). SDS-PAGE and WB were employed to assess the presence of Nf in the isolated fractions prepared by UC or captured by SEP. SDS-PAGE analysis of these fractions showed considerably different protein migration patterns (Fig. 2A), indicating that the two methodologies enrich different sets of aggregates and proteins.

We next examined the enriched samples for the presence and migration patterns of neurofilaments. In the pellets after UC, as show in Fig. 1, all three Nf isoforms were detectable (Fig. 2B). NfH was present as a high, SDS-stable, MW band migrating above the 460 kDa marker (Fig. 2B, upper panel, arrow). In comparison, WB analysis of NfH in PPS prior to ultracentrifugation showed only the expected 238 kDa band with no higher molecular weight bands (Fig. 1). This suggests that UC-processing successfully enriches NfH-containing macromolecular formations otherwise not detectable, most likely due to low concentration in un-processed plasma. We detected three forms of NfM in the aggregate fraction (Fig. 2B, middle panel), with the most intense migrating at the same MW (117 kDa) as observed in non-enriched PPS (Fig. 1). In common with the un-processed PPS, NfL appeared only as a possibly truncated product at around 30 kDa (Fig. 2B, lower panel and Fig. 1).

WB analysis of SEP-captured samples showed several NfH-reactive bands but none of these migrated above the expected 238 kDa band seen in the un-processed samples (Fig. 2C, upper panel and Fig. 1). Similarly, NfM migrated at various MW, including above 200 kDa (Fig. 2C, middle panel). The most intense of these anti-NfM bands appeared at around 55 kDa and was also observed in the aggregate fraction obtained using UC, possibly representing an immunoreactive fragment of the full-size protein. No NfL band was observed in the SEP-captured fraction, either at the expected or at any other MW (Fig. 2C, lower panel).

### 3.4 LC-MS/MS proteomics of UC and SEP fractions

**Fig. 3:**
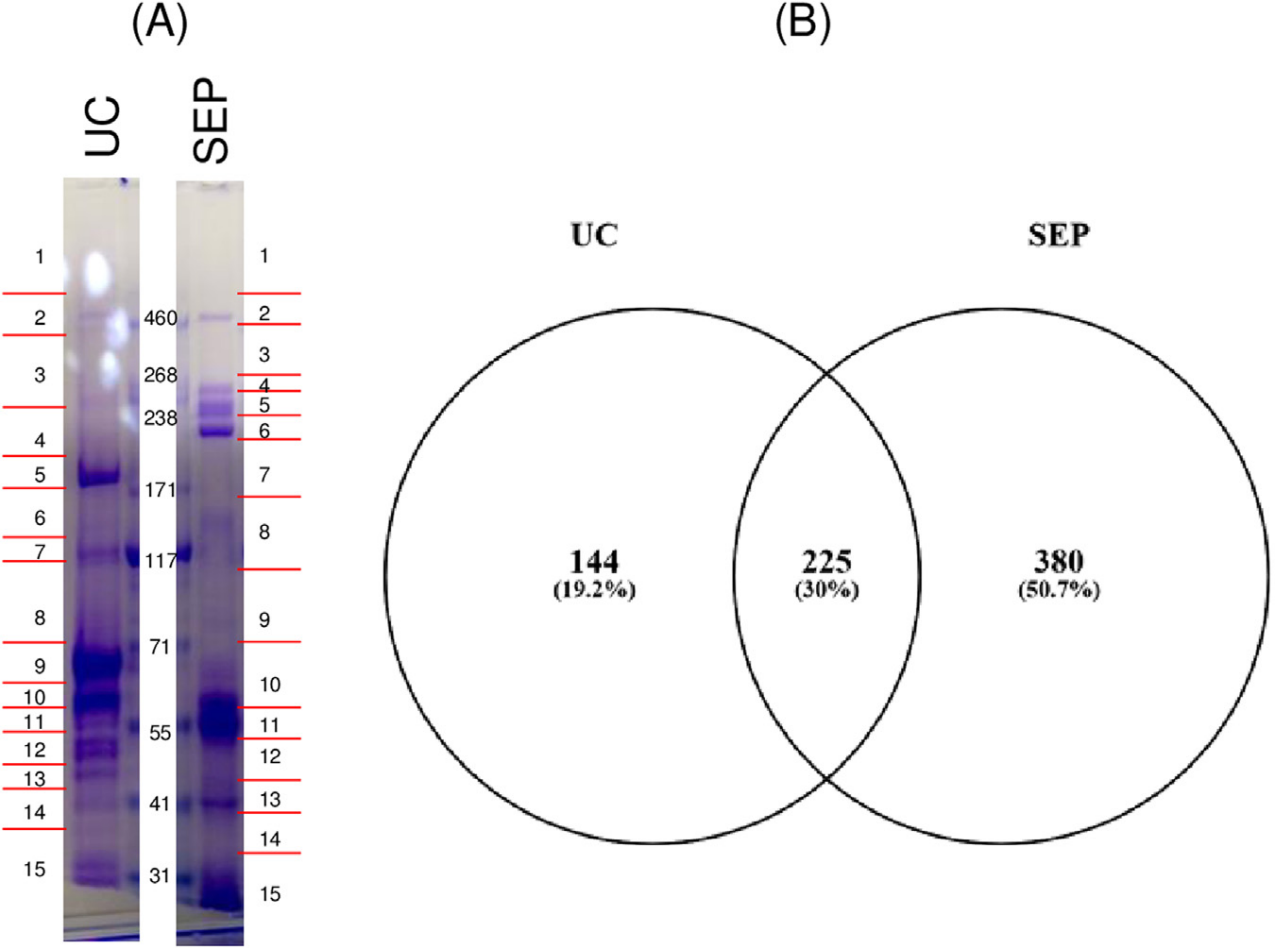
UC and SEP aggregates fractions for LC-MS/MS analysis. (A) SDS-PAGE showing gel bands dissected for in-gel Trypsin digestion for LC-MS/MS (a total of 15 for both UC and SEP) (same picture shown in A); (B) Protein Identification performed with Proteome Discoverer 1.4 generated 369 and 605 proteins unique to UC and SEP respectively; 225 were shared by the two extraction methods (2 peptides for protein as minimum and a peptide count only in the top scored proteins were utilized). UC: ultracentrifugation; SEP: Seprion extraction pads.

We next used proteomics to compare the protein contents of the enriched fractions isolated from PPS by UC and SEP. We separated comparable amount of total protein from these samples by SDS-PAGE (Fig. 3A). Each lane was divided into 15 individual sections and subjected to in-gel tryptic digestion followed by LC-MS/MS analysis. We identified 369 and 605 protein groups for UC_PPS and SEP_PPS respectively (Fig. 3; identified protein file). Nf proteins were not detected using LC-MS/MS in neither the UC_PPS nor in the SEP_PPS, possibly due to the occurrence of complex PTMs and the low abundance of Nf proteins, (see Discussion for more details). Approximately a third (30%, 225/749) of the identified proteins were detected by both extraction methodologies (Fig. 3B). The larger number of proteins observed in the SEP_PPS analysis was not due to unequal sample loading, as UC_PPS and SEP_PPS fractions had the same number of spectra acquired from the instrument and a comparable ID rate (Peptide Spectrum Matches (PSMs)/total spectra; Table 2). However, UC_PPS appeared to have a higher coverage and number of PSMs per protein group, suggesting that this extraction method was more selective in the enrichment of specific proteins. The UC_PPS and SEP_PPS shared proteins had higher protein coverage and PSMs counts than those seen in either of the individual enrichment methods, suggesting that these shared proteins were more abundant in plasma (Table 2). The lists of shared proteins and of proteins specific to each aggregate-enrichment method were analysed for enrichment of cellular component (CC) and KEGG [32] pathway terms, using PANTHER [28] and Webgestalt [29] respectively.

**Table 2:** MS analysis of enriched aggregates: main features of the protein groups identified using Proteome Discoverer 1.4. Data are shown for the protein groups identified by ultracentrifugation only (UC), by Seprion Pads only (SEP), or “shared” by the two enrichment methods. The shared fraction included only the protein isoforms in common and not the parental proteins: coverage values are normally distributed and the mean was used for comparison.

**fraction**	**total spectra**	**total PSMs**	**ID rat%**	**protein groups**	**Coverage%/ protein group (mean)**	**#Unique Peptides/ protein group (median)**	**#Peptides/ protein group (median)**	**#PSMs/ protein group (median)**
UC	215,993	35,779	16.56	371	31.2%	5.0	6.0	12.0
SEP	210,875	34,865	16.53	609	25.6%	5.0	6.0	8.0
UC (shared)				209	34.7%	7.0	10.0	28.0
SEP (shared)				209	35.3%	8.0	10.0	21.0

#### 3.4.1 UC and SEP-shared proteomic data analysis

**Fig. 4:**
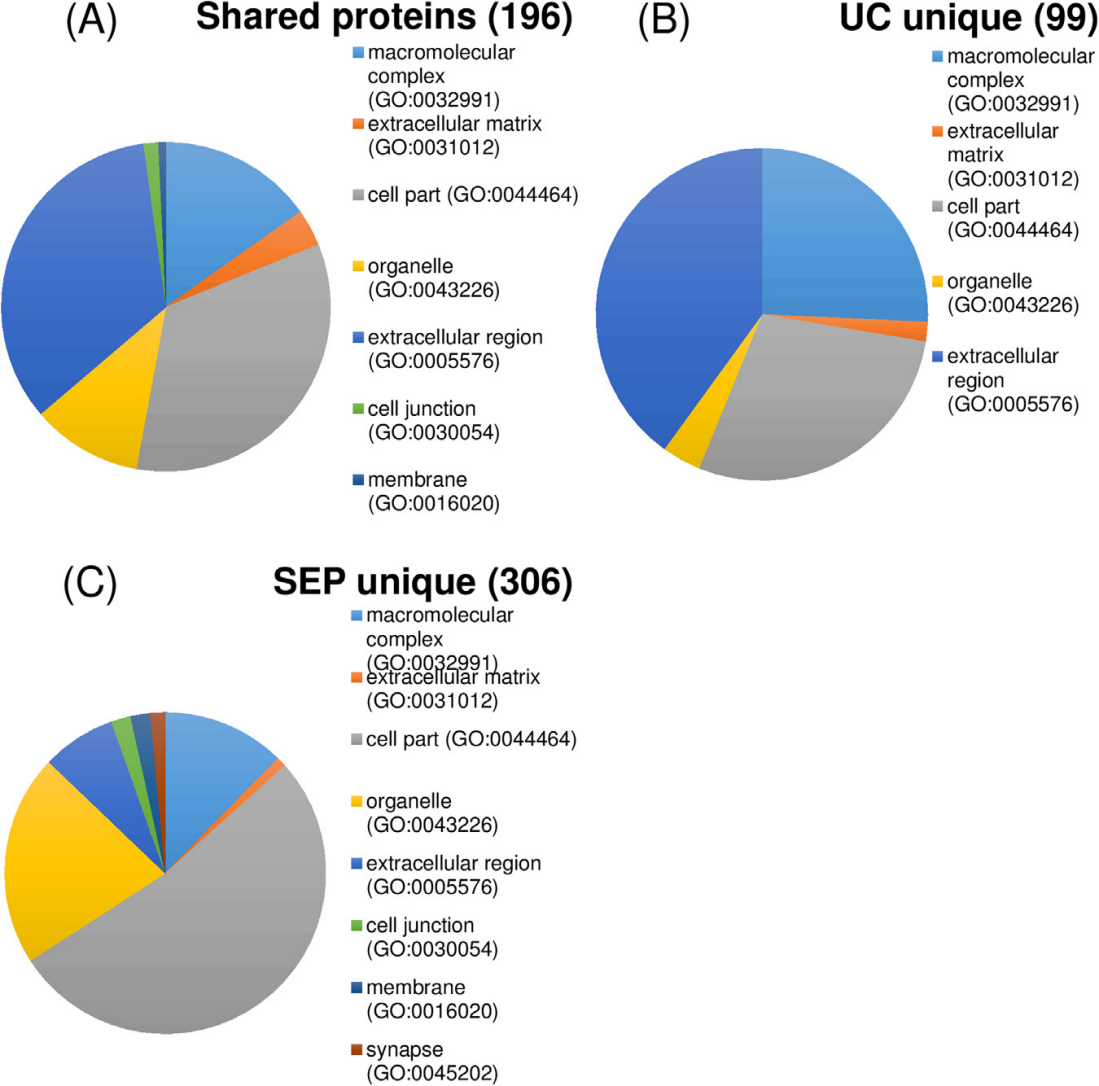
Bioinformatics analysis of the MS proteomics data of the aggregate extraction products obtained using Protein Analysis THrough Evolutionary Relationships (PANTHER). Pie charts representing Cellular Component (CC) category composition in (A) shared proteins between UC and SEP, (B) unique proteins detected by UC and (C) by SEP. In brackets, the number of proteins identified by PANTHER for each group.

PANTHER classification analysis of the 225 entries shared by UC and SEP identified 196 proteins, with 138 hits within the Cellular Component (CC) (Fig. 4A). Of these, 47 proteins (34.1% of the total hits) were classified as Cell Part, the majority being intracellular (35, 67.3%), and extracellular region (47, 34.1%). The other two main CC were macromolecular complexes (15.2%) and organelles (10.9%). Ten proteins within the organelles category were classified as cytoskeletal proteins. The top 10 enriched features obtained using KEGG pathway analysis of the shared fraction are shown in Table 3. Most pathways were linked to infection and immune response (Staphylococcus aureus infection, pertussis, systemic lupus erythematosus, pathogenic Escherichia coli infection, complement, platelet activation) with the remaining related to cell migration (adhesion), extra-cellular matrix and prion disease. As highlighted by PANTHER analysis, the cellular components involved in these pathways were related with extracellular region and cell part terms.

**Table 3:** Top 10 enriched KEGG pathways in the pool of UC and SEP shared proteins. PValue: p-value from hypergeometric test; FDR: False Discovery Rate from Bonferroni Hypothesis.

**Geneset**	**Pathway description**	**Number of reference genes in the category**	**Number of genes in the user gene list and also in the category**	**Expected number in the category**	**Ratio of enrichment**	**PValue**	**FDR**
hsa04610	Complement and coagulation cascades - Homo sapiens (human)	79	40	1.23	32.42	0.00E+00	0.00E+00
hsa05150	Staphylococcus aureus infection - Homo sapiens (human)	56	13	0.87	14.86	1.72E−12	2.60E−10
hsa05020	Prion diseases - Homo sapiens (human)	35	8	0.55	14.63	4.53E−08	4.58E−06
hsa05133	Pertussis - Homo sapiens (human)	76	10	1.19	8.42	2.30E−07	1.74E−05
hsa04510	Focal adhesion - Homo sapiens (human)	203	14	3.17	4.42	2.78E−06	1.69E−04
hsa05322	Systemic lupus erythematosus - Homo sapiens (human)	135	11	2.11	5.22	7.20E−06	3.64E−04
hsa05130	Pathogenic Escherichia coli infection - Homo sapiens (human)	55	7	0.86	8.15	2.04E−05	8.85E−04
hsa04145	Phagosome - Homo sapiens (human)	154	11	2.41	4.57	2.52E−05	9.56E−04
hsa04512	ECM-receptor interaction - Homo sapiens (human)	82	7	1.28	5.47	2.71E−04	9.13E−03
hsa04611	Platelet activation - Homo sapiens (human)	122	8	1.91	4.20	6.03E−04	1.83E−02

#### 3.4.2 UC-specific proteomic data analysis

PANTHER generated 99 Cellular Component proteins (105 hits) from the LC-MS/MS analysis of the UC_PPS fraction (Fig. 4B). Of these, 40% were classified as derived from the extracellular region, 28.6% as cell part and 25.7% as macromolecular complex, while the rest belonged to extracellular matrix (1.9%) and organelles (3.8%). 26.7% of the cell part component was made of intracellular proteins and 73.30% was plasma membrane, while 20% of the organelle component was part of the cytoskeleton. Functional analysis by KEGG (Table 4) revealed a significant representation of features involved in endocrine, metabolic and cell-signalling regulation (PPAR, thyroid hormone synthesis, lysine degradation, carbon metabolism, citrate-TCA cycle) and pathways specific to ECM-receptor interaction, phagosome and adipocytokine signalling.

**Table 4:** Top 10 enriched KEGG pathways for UC_PPS. PValue: p-value from hypergeometric test; FDR: False Discovery Rate from Bonferroni Hypothesis.

**Geneset**	**Pathway description**	**Number of reference genes in the category**	**Number of genes in the user gene list and also in the category**	**Expected number in the category**	**Ratio of enrichment**	**PValue**	**FDR**
hsa00020	Citrate cycle (TCA cycle) - Homo sapiens (human)	30	3	0.13	23.26	2.69E−04	0.06
hsa04512	ECM-receptor interaction - Homo sapiens (human)	82	4	0.35	11.35	3.85E−04	0.06
hsa04145	Phagosome - Homo sapiens (human)	154	4	0.66	6.04	4.00E−03	0.40
hsa01200	Carbon metabolism - Homo sapiens (human)	114	3	0.49	6.12	1.25E−02	0.95
hsa05144	Malaria - Homo sapiens (human)	49	2	0.21	9.50	1.85E−02	1.00
hsa05130	Pathogenic Escherichia coli infection - Homo sapiens (human)	55	2	0.24	8.46	2.30E−02	1.00
hsa00310	Lysine degradation - Homo sapiens (human)	59	2	0.25	7.89	2.63E−02	1.00
hsa04920	Adipocytokine signalling pathway - Homo sapiens (human)	70	2	0.30	6.65	3.60E−02	1.00
hsa03320	PPAR signalling pathway - Homo sapiens (human)	72	2	0.31	6.46	3.79E−02	1.00
hsa04918	Thyroid hormone synthesis - Homo sapiens (human)	74	2	0.32	6.29	3.99E−02	1.00

#### 3.4.3 SEP-specific proteomic data analysis

From the 380 unique proteins obtained by LC-MS/MS analysis of the SEP fraction, PANTHER identified 306 proteins generating 202 hits within Cellular Component (Fig. 4C). The main component was cell part (52.5%), of which 79.7% were intracellular and 18% were plasma membrane proteins, followed by organelle (21.3%) and macromolecular complex (12.4%) components, while extracellular region, cell junction, membrane, synapse and extracellular matrix accounted for 13.9% of the total hits. Most of the organelle sub-component proteins were related to the cytoskeleton (53.2%). KEGG functional analysis identified features related to immune activation (chemokine signalling pathway, focal adhesion, shigellosis, platelet activation) and to cell function and structure (tight junction, regulation of actin cytoskeleton, vascular smooth muscle contraction, Table 5). All pathways showed enrichments with a FDR < 0.1.

**Table 5:** Top 10 enriched KEGG pathways for SEP_PPS. PValue: p-value from hypergeometric test; FDR: False Discovery Rate from Bonferroni Hypothesis.

**Geneset**	**Pathway description**	**Number of reference genes in the category**	**Number of genes in the user gene list and also in the category**	**Expected number in the category**	**Ratio of enrichment**	**PValue**	**FDR**
hsa04810	Regulation of actin cytoskeleton - Homo sapiens (human)	216	27	5.23	5.16	1.11E−12	3.37E−10
hsa04611	Platelet activation - Homo sapiens (human)	122	16	2.95	5.42	3.05E−08	3.67E−06
hsa05131	Shigellosis - Homo sapiens (human)	65	12	1.57	7.62	3.64E−08	3.67E−06
hsa04270	Vascular smooth muscle contraction - Homo sapiens (human)	121	15	2.93	5.12	1.80E−07	1.24E−05
hsa04921	Oxytocin signalling pathway - Homo sapiens (human)	159	17	3.85	4.42	2.36E−07	1.24E−05
hsa04144	Endocytosis - Homo sapiens (human)	260	22	6.30	3.49	2.46E−07	1.24E−05
hsa05130	Pathogenic Escherichia coli infection - Homo sapiens (human)	55	10	1.33	7.51	6.07E−07	2.63E−05
hsa04530	Tight junction - Homo sapiens (human)	139	14	3.37	4.16	5.87E−06	2.23E−04
hsa04510	Focal adhesion - Homo sapiens (human)	203	17	4.92	3.46	7.27E−06	2.45E−04
hsa04062	Chemokine signalling pathway - Homo sapiens (human)	187	16	4.53	3.53	1.04E−05	3.16E−04

### 3.5 Pro-aggregation proteins

We then interrogated the Curated Protein Aggregation Database (CPAD) database [33], to evaluate whether proteins with known pro-aggregating properties were in our LC-MS/MS datasets. Of the 27 human pro-aggregating proteins in the database, four were found in both the UC_PPS and SEP_PPS proteomes (Gelsolin, Apolipoprotein A-I, Kerato-epithelin, Lysozyme C) and two uniquely in the SEP_PPS fraction (Semenogelin-1; Lactoferrin).

## 4 Discussion

The detection of Nf in biological fluids is one of the most promising biomarkers of neurodegeneration, but its use is complicated by the apparent non-linearity of detection in serial dilutions. We have previously suggested that this caveat is the result of Nf, in particular the heavy chain, being sequestered into stable protein aggregates, masking epitopes from immunodetection. Here we show that Nf are part of high-molecular weight hetero-aggregates in blood. Such circulating protein aggregates may convey information on proteinopathies linked to specific organ pathologies, thus functioning as a novel source of disease biomarkers.

Most of the experimental work produced so far on the detection and quantification of protein aggregates has been undertaken in tissues and cell systems [34], [35], [36], [37]. In the field of neurodegeneration, for example, protein aggregates are generally initiated by specific “seed” proteins such as Tau and beta-amyloid. These aggregates are normally isolated using ultracentrifugation, membrane filter assays and magnetic‐bead immune-affinity pull downs, with little attention for the wide range of other co‐aggregated proteins [34], [35], [36], [37]. The emerging evidence that different stages of protein aggregation can be also seen in biofluids and the idea that this phenomenon could somehow reproduce brain pathology has moved this field of investigation towards the characterization of the fibrillary and aggregated state of proteins as potential disease biomarkers [15], [38]. Therefore, the development of sound methodologies for the isolation and characterization of circulating protein aggregates from biofluids is critical not only for diagnostic purposes but also for monitoring the efficacy of anti-aggregation therapies currently being developed.

Our study indicates that the choice of methodology for extraction of circulating aggregates is critical to the information likely to be obtained. Sedimentation and binders exploit different physical properties in the enrichment process, with the latter based on affinity for proteins involved in different levels of aggregation [39]. Our data show that Seprion technology isolates a higher number of proteins, an observation that may be explained by Seprion's broader affinity to any conformational change, which affect protein-protein interactions. As a result, it is less certain that proteins identified following Seprion enrichment are truly part of hetero-aggregates and not simply co-isolated disordered monomers or low order multimers. It is particularly interesting to note that the two separation methods yielded different profiles of neurofilament content. The different molecular weights for NfH and NfM recovered by both methods and the lack of NfL detection in the Seprion enriched-material suggest that these methods may sample different molecular entities. This phenomenon may be driven by post-translational modifications (PTMs) of neurofilaments or of other proteins which ultimately affect their binding to Seprion. Whilst such investigation is beyond the purpose of the current study, it would possible to perform enhanced search of the mass spectrometer raw data files to include multiple PTM's. UC separation on the other hand is relying only on density and may thus predominantly capture large and dense aggregates and not the initial, lower molecular weight seeding species. Nevertheless, UC separation seems to be more stringent, generating a smaller and possibly less complex proteome, while acquiring, on average, more spectra for the same peptide. This shows that although the UC enrichment method coupled with MS analysis produces a smaller share of information in terms of number of proteins, it may also generate less false positive information in protein identification compared to Seprion. Seprion methodology may be used in experimental settings where exploratory studies are performed to identify a broad range of altered protein targets which escape proteostatic control [15].

MS proteomics shows that aggregates extracted from healthy individual plasma samples using UC and SEP mostly harbour cellular components and extracellular matrix proteins. Functionally, both extraction techniques identify proteins networked within inflammatory processes, particularly complement factors, along with phagosome and prion-related proteins. However, qualitative differences have emerged in the protein pools of the aggregates extracted using the two methodologies. UC aggregates are specifically enriched with proteins involved in endocrine, metabolic and cell-signalling regulation, while SEP aggregates show enrichment in proteins involved in cellular processes and in the immune system. Overall, it could be speculated that these hetero-complexes contain biological factors that are essential in maintaining homeostasis, like those regulating inflammation and metabolism, while they may also act as sinks for the sequestration of other proteins whose concentration increases with the development of pathological conditions.

In this study, we have confirmed by Western blot that Nf are included in high molecular weight complexes in plasma as previously anticipated [21], [22]. Our data also strengthen the hypothesis that NfH is more likely to be sequestered into metastable, circulating aggregates while smaller and perhaps less complex Nf isoforms like NfL are not, in line with the reported NfL linearity when tested in dilution curves compared to the hook effect seen with NfH [24]. However, despite the immunoblotting detection of Nf in both UC and SEP fractions, we did not identify Nf in either fraction using MS. As the plasma used in this study was derived from healthy individuals, it is likely that Nf levels would have been lower than in established neurodegenerative diseases such as ALS. Nf low levels escaping detection by standard MS proteomics may also be related to the prevalence of circulating protein aggregates non containing Nf which can mask the detection of NCH, likely to be far less represented in healthy individuals. It is also possible that NfH in circulating aggregates may not be readily digestible [15] using trypsin, with resulting peptides that are too big for the mass-to-charge range that has been selected for our bottom-up proteomic approach. To this end, it may be worth considering whether Nf found in circulating protein complexes may have a modified protease resistance which condition their propensity to form aggregates. Furthermore, all Nf isoforms are subjected to a variety of post-translational modifications [40], [41] which influence the detection of tryptic peptides without employing specialized strategies. The KSP (Lysine-Serine-Proline) repeats positioned in the Nf tail are often the subjects of complex PTMs and therefore, may escape detection when MS-based proteomics is applied, particularly when using trypsin, LysN or LysC. To test this hypothesis, we re-submitted unmatched spectra to search against a Nf-only sequence database. Searching was performed for tryptic peptides with variable phosphorylation of serine, threonine and tyrosine residues and N-Glycosylation, two major post-translational modifications. Whilst we could match several spectra to Nf, using this approach, the statistical analysis was inherently weakened by the relatively small size of the created database. Further optimisation of the analysis may be undertaken in the future to convincingly identify Nf in biofluids by MS-based proteomics.

Our experimental approach has so far targeted the protein component of complexes which may be heterogeneous, as the presence of nucleic acids and lipids within these formations cannot be excluded. This is particularly important in neurodegenerative conditions where the presence of both RNA and RNA–binding proteins have been shown to mediate protein aggregation/phase transition and to be required for recruitment into stress granules [3]. It should also be noted that the potential of the analytical approach described in this paper will have to be further evaluated in a pathological context, where the hypothesis to be tested is whether the circulating aggregate could function as a reporter of pathologies like neurodegenerative disorders and whether it plays a role in the processing and/or transport of centrally produced, conformationally altered proteins. As Nf are found in biofluids, it is tempting to speculate that other brain-specific proteins which are also aggregation-prone may follow the same path, enhancing protein aggregation in the fluid phase. While not identical to aggregates found in brain under pathological conditions, these circulating protein complexes may bear some of the features of these neurological formations, providing a useful test-bed for the development of future phase-specific biomarkers.

While UC and Seprion-based technologies may be the methods of choice for aggregates separation, they may also present different sensitivities for the selection of disease-specific proteins which are packaged within circulating hetero-complexes.

## 5 Conclusions

In this study, we have tested different modalities for the isolation from plasma of NCH and obtained a detailed characterization of their proteome. As for Nf, we believe that NCH composition may provide a biomarker for several neurodegenerative diseases, particularly amyotrophic lateral sclerosis (ALS), where Nf levels in biofluids have a strong prognostic value [22], [25], [42], [43]. We also postulate that any organ-specific or systemic pathological state involving protein catabolism or homeostasis, may promote the formation of unique aggregates in biofluids providing the ground to uncover novel disease biomarkers for clinical monitoring.

## References

[1] Ross C.A., Poirier M.A. (2005). What is the role of protein aggregation in neurodegeneration?. Nat. Rev. Mol. Cell Biol..

[2] Yang H., Hu H.Y. (2016). Sequestration of cellular interacting partners by protein aggregates: implication in a loss-of-function pathology. FEBS J..

[3] Brangwynne C.P. (2011). Soft active aggregates: mechanics, dynamics and self-assembly of liquid-like intracellular protein bodies. Soft Matter.

[4] David D.C. (2010). Widespread protein aggregation as an inherent part of aging in C. elegans. PLoS Biol..

[5] Kaganovich D., Kopito R., Frydman J. (2008). Misfolded proteins partition between two distinct quality control compartments. Nature.

[6] Wallace E.W. (2015). Reversible, specific, active aggregates of endogenous proteins assemble upon heat stress. Cell.

[7] Walther D.M. (2015). Widespread proteome remodeling and aggregation in aging C. elegans. Cell.

[8] Rubinsztein D.C. (2006). The roles of intracellular protein-degradation pathways in neurodegeneration. Nature.

[9] Buchberger A., Bukau B., Sommer T. (2010). Protein quality control in the cytosol and the endoplasmic reticulum: brothers in arms. Mol. Cell.

[10] Lim J., Yue Z. (2015). Neuronal aggregates: formation, clearance, and spreading. Dev Cell.

[11] Li W. (2016). Antibody aggregation: insights from sequence and structure. Antibodies.

[12] Sahin E. (2012). Aggregation and pH-temperature phase behavior for aggregates of an IgG2 antibody. J. Pharm. Sci..

[13] Kolhe P., Amend E., Singh S.K. (2010). Impact of freezing on pH of buffered solutions and consequences for monoclonal antibody aggregation. Biotechnol. Prog..

[14] Finn T.E. (2012). Serum albumin prevents protein aggregation and amyloid formation and retains chaperone-like activity in the presence of physiological ligands. J. Biol. Chem..

[15] Xia K. (2016). Increased levels of hyper-stable protein aggregates in plasma of older adults. Age.

[16] Kim S., An S.S. (2016). Role of p53 isoforms and aggregations in cancer. Medicine.

[17] Blokhuis A.M. (2013). Protein aggregation in amyotrophic lateral sclerosis. Acta Neuropathol..

[18] Gentil B.J., Tibshirani M., Durham H.D. (2015). Neurofilament dynamics and involvement in neurological disorders. Cell Tissue Res..

[19] Grant P., Pant H.C. (2000). Neurofilament protein synthesis and phosphorylation. J. Neurocytol..

[20] Slawson C., Hart G.W. (2003). Dynamic interplay between O-GlcNAc and O-phosphate: the sweet side of protein regulation. Curr. Opin. Struct. Biol..

[21] Lu C.H. (2011). A method to solubilise protein aggregates for immunoassay quantification which overcomes the neurofilament "hook" effect. J. Neurosci. Methods.

[22] Lu C.H. (2015). Plasma neurofilament heavy chain levels and disease progression in amyotrophic lateral sclerosis: insights from a longitudinal study. J. Neurol. Neurosurg. Psychiatry.

[23] Szaro B.G., Strong M.J. (2010). Post-transcriptional control of neurofilaments: new roles in development, regeneration and neurodegenerative disease. Trends Neurosci..

[24] Gaiottino J. (2013). Increased neurofilament light chain blood levels in neurodegenerative neurological diseases. PLoS One.

[25] Lu C.H. (2015). Neurofilament light chain: a prognostic biomarker in amyotrophic lateral sclerosis. Neurology.

[26] Bendotti C. (2004). Activated p38MAPK is a novel component of the intracellular inclusions found in human amyotrophic lateral sclerosis and mutant SOD1 transgenic mice. J. Neuropathol. Exp. Neurol..

[27] A. Lane, et al., Polymeric ligands with specificity for aggregated prion proteins. patent, 2003. PCT/GB03/00858.

[28] Thomas P.D. (2003). PANTHER: a browsable database of gene products organized by biological function, using curated protein family and subfamily classification. Nucleic Acids Res..

[29] Zhang B., Kirov S., Snoddy J. (2005). WebGestalt: an integrated system for exploring gene sets in various biological contexts. Nucleic Acids Res..

[30] Noble W.S. (2009). How does multiple testing correction work?. Nat. Biotechnol..

[31] Sathasivam K. (2010). Identical oligomeric and fibrillar structures captured from the brains of R6/2 and knock-in mouse models of Huntington's disease. Hum. Mol. Genet..

[32] Kanehisa M., Goto S. (2000). KEGG: kyoto encyclopedia of genes and genomes. Nucleic Acids Res..

[33] Thangakani A.M. (2016). CPAD, curated protein aggregation database: a repository of manually curated experimental data on protein and peptide aggregation. PLoS One.

[34] Scherzinger E. (1997). Huntingtin-encoded polyglutamine expansions form amyloid-like protein aggregates in vitro and in vivo. Cell.

[35] Wanker E.E. (1999). Membrane filter assay for detection of amyloid-like polyglutamine-containing protein aggregates. Methods Enzymol..

[36] Chang E., Kuret J. (2008). Detection and quantification of tau aggregation using a membrane filter assay. Anal. Biochem..

[37] Ayyadevara S. (2016). Proteins that mediate protein aggregation and cytotoxicity distinguish Alzheimer's hippocampus from normal controls. Aging Cell.

[38] Dekker A.D. (2017). Cerebrospinal fluid biomarkers for Alzheimer's disease in Down syndrome. Alzheimers Dement..

[39] Benn C.L. (2009). Genetic knock-down of HDAC7 does not ameliorate disease pathogenesis in the R6/2 mouse model of Huntington's disease. PLoS One.

[40] Betts J.C. (1997). Identification of phosphorylation sites on neurofilament proteins by nanoelectrospray mass spectrometry. J. Biol. Chem..

[41] Dong D.L. (1993). Glycosylation of mammalian neurofilaments. Localization of multiple O-linked N-acetylglucosamine moieties on neurofilament polypeptides L and M. J. Biol. Chem..

[42] Gaiani A. (2017). Diagnostic and prognostic biomarkers in amyotrophic lateral sclerosis: neurofilament light chain levels in definite subtypes of disease. JAMA Neurol.

[43] Xu Z. (2016). Neurofilaments as biomarkers for amyotrophic lateral sclerosis: a systematic review and meta-analysis. PLoS One.

